# Post-TB sequelae in adolescent pulmonary TB survivors

**DOI:** 10.5588/ijtldopen.24.0039

**Published:** 2025-01-01

**Authors:** C. Cesilia, F.R. Rinawan, P. Santoso, H.M. Nataprawira

**Affiliations:** ^1^ Division of Respirology, Department of Child Health, Faculty of Medicine, Universitas Padjadjaran/Dr Hasan Sadikin General Hospital, Bandung, Indonesia;; ^2^ Faculty of Medicine, University of Riau, Pekanbaru, Indonesia;; ^3^ Department of Public Health, Faculty of Medicine, Universitas Padjadjaran, Bandung, Indonesia;; ^4^ Division of Pulmonology and Critical Care Medicine, Department of Internal Medicine, Faculty of Medicine, Universitas Padjadjaran/Dr Hasan Sadikin General Hospital, Bandung, Indonesia.

**Keywords:** adolescent, children, post-tuberculosis lung disease, tuberculosis

## Abstract

**OBJECTIVE:**

To compare the persistent clinical symptoms, chest X-ray (CXR), spirometry and echocardiography results in adolescent survivors of drug-susceptible (DS) and drug-resistant (DR) pulmonary TB (PTB).

**METHODS:**

This retrospective cohort study was conducted in 52 adolescent PTB survivors. We compared persistent clinical symptoms, CXR, spirometry and echocardiography in DS-TB and DR-TB survivors. χ^2^ test was used to compare groups (*P* < 0.05 significant), logistic regression analysis was used to identify risk factors, and Many-Factor Rasch Measurement Version Facets 3.86.0 was used to assess the multi-rater agreement of CXR.

**RESULTS:**

Significant differences were found in persistent clinical symptoms (higher in DS-TB survivors), CXR, and spirometry abnormalities (higher in DR-TB survivors). The echocardiography revealed a low pulmonary hypertension probability in both groups. Adolescent PTB survivors with comorbidity have 1.5 times the risk of developing pulmonary sequelae compared to non-sequelae.

**CONCLUSION:**

Pulmonary sequelae in adolescent PTB survivors were documented in both DS and DR-TB. Monitoring after treatment completion was necessary for PTB survivors, especially in adolescents.

## RÉSUMÉ

**OBJECTIF:**

Comparer les symptômes cliniques persistants, les résultats de la radiographie pulmonaire (CXR), les données de la spirométrie ainsi que ceux de l'échocardiographie chez les adolescents ayant survécu à une TB pulmonaire (PTB) sensible aux médicaments (DS-TB) et résistante aux médicaments (DR-TB).

**MÉTHODES:**

Cette étude de cohorte rétrospective a été menée auprès de 52 adolescents ayant survécu à la PTB. Nous avons comparé les symptômes cliniques persistants, le CXR, la spirométrie et l'échocardiographie chez les survivants de la DS-TB et de la DR-TB. Le test du χ^2^ a été appliqué pour comparer les différents groupes (*P* < 0,05 étant considéré comme significatif), tandis que l'analyse de régression logistique a permis d'identifier les facteurs de risque. De plus, la version 3.86.0 de Many-Factor Rasch Measurement a été utilisée pour évaluer l'accord multilatéral des résultats de la CXR.

**RÉSULTATS:**

Des variations notables ont été observées concernant la persistance des symptômes cliniques, qui sont plus fréquents chez les survivants de la DS-TB. En revanche, les anomalies détectées par la CXR et la spirométrie sont plus prononcées chez les survivants de la DR-TB. L'échocardiographie a révélé une faible probabilité d'hypertension pulmonaire dans les deux groupes étudiés. De plus, les adolescents ayant survécu à la PTB avec des comorbidités présentent un risque 1,5 fois supérieur de développer des séquelles pulmonaires par rapport à ceux qui n'en souffrent pas.

**CONCLUSION:**

Des séquelles pulmonaires ont été observées chez les adolescents ayant survécu à la PTB, tant dans le groupe des patients atteints de DS-TB que dans celui des patients atteints de DR-TB. Il est essentiel d'assurer un suivi après la fin du traitement pour ces survivants, en particulier pour les adolescents.

Post-TB sequelae are gaining increasing attention in clinical and public health domains. Post-TB sequelae are defined as persistent respiratory symptoms, radiological abnormalities, lung function impairment, and vascular disease after completion of adequate therapy,^1,2^ which have adverse impacts on the growth and development of children and can lead to lasting functional consequences persisting even into adulthood.^3^ A systematic review by Ivanova et al. noted that in the majority of the studies included, approximately 10–15% of pulmonary TB (PTB) survivors had severe lung impairment.^4^ Impairments in lung functions, presenting with abnormal forced expiratory volume in 1 second (FEV_1_), were associated with a greater risk of death from cardiovascular causes and a higher risk of frequent hospitalisation for respiratory issues.^5^

Our study aimed to compare the persistent clinical symptoms, chest X-ray (CXR), spirometry, and echocardiography results of adolescents with drug-susceptible (DS) and drug-resistant (DR) TB.

## METHODS

### Study design and population

A retrospective cohort study was performed with the study population of adolescent PTB survivors (aged 10−18 years at the time of PTB diagnosis) treated at Dr Hasan Sadikin General Hospital, Bandung, Indonesia, from January 2019 to December 2022. Participants were selected based on consecutive sampling and randomised using MS Excel (Microsoft, Seattle, WA, USA). The minimum sample size targeted was 32 per group, determined using the formula for comparing two proportions of dichotomous variables between DR- and DS-TB. The study assumes a significance level of α = 5% (Zα = 1.96; two-tailed) and a power of 80% (Zβ = 0.84).^6^ Variables (sex, age, nutritional status, monthly family income, vaccination, smoking habits per day, comorbidity, TB contact, type of TB, episode of TB treatment, bacteriological status, duration from onset to initial therapy, and duration from completed treatment to evaluation examination) were collected from the DR-TB register of the Division of Respirology, Department of Child Health, Faculty of Medicine, Universitas Padjadjaran, Bandung, Indonesia; and the National Tuberculosis Information System.

### Inclusion and exclusion criteria

Inclusion criteria were adolescents (aged 10–18 years at the time of PTB diagnosis) with a previous history of completed treatment or cure of PTB. Exclusion criteria consist of participants with symptoms of acute respiratory infection (ARI) during the examination and positive SARS-CoV-2 antigen swab results; contraindicated for a spirometry test, uncooperative during testing; and unable to be followed-up/contacted for participation.^7^

### Operational definitions

Diagnosis of DS- and DR-TB was based on WHO criteria for TB in children and adolescents.^8^ In this study, we defined pulmonary sequelae as any abnormalities in one or more persistent clinical symptoms, CXR, spirometry, and echocardiography in PTB survivors. Persistent respiratory symptoms are clinical respiratory symptoms that persist for more than 4 weeks or recurring (>2 episodes within 6 months).^9,10^ The CXR abnormalities referred to radiological findings related to parenchymal or airway damage,^9^ with final diagnosis made when at least 2 of the three consultant radiologists had reached a consensus (blinded to the study). Spirometry was performed using a MIR SPIROLAB CODE 46906 (Medical International Research, Rome, Italy) spirometer. A certified paediatric respirology trainee demonstrated the spirometry procedure for each participant. Measurements were taken in three repeatable and acceptable forced vital capacity (FVC) manoeuvres.^7^ Spirometry results were interpreted by a paediatric respirology and an internist pulmonologist consultant (blinded to the study). The results were classified based on the cut-offs recommended in the 2023 Global Initiative for Chronic Obstructive Lung Disease (GOLD) guidelines as follows:^11^ 1) obstructive syndromes (FEV_1_/FVC <70% with FEV_1_ of predicted >70%): mild (FEV_1_ >80%); moderate (FEV_1_ 50–80%); severe (30–50%); very severe (FEV_1_ <30%); and 2) restrictive syndromes (FEV_1_/FVC was normal with low FVC): mild (FVC <80%); moderate (FVC <60%); severe (FVC <50%).

Echocardiography was performed using Philips EPIQ 5 Ultrasound Machine (Philips, Amsterdam, The Netherlands) to measure tricuspid regurgitation velocity (TRV) for PH risk assessment by three paediatric cardiology consultants (blinded to the study). Risk for PH was determined according to TRV and clinical signs as follows: low (peak TRV ≤2.8 m/s *or* unmeasured without other signs of PH based on echocardiography); intermediate (peak TRV ≤2.8 m/s or unmeasured with other signs of PH *or* peak TRV 2.9−3.4 m/s without signs of PH); and high (peak TRV 2.9−3.4 m/s with other signs of PH *or* peak TRV >3.4 m/s).^12^

### Ethical clearance

This study adhered to the Helsinki declaration. Informed consent was obtained from parents/guardians and participants; ethical clearance (LB.02.01/X.6.5/108/2023) and hospital permission (DP.04.03/X.2.2.1/7137/2023) were obtained from the Ethical Committee of the Dr. Hasan Sadikin General Hospital, Bandung, Indonesia, before the study.

### Data analysis

Descriptive statistics include frequency (percentage) or median. χ^2^ test was used to compare continuous variables (categorised prior). Logistic regression analyses were conducted among pulmonary sequelae after PTB groups. Odds ratios (ORs) were used to estimate the association between pulmonary sequelae after TB and variables. The multi-rater agreement value was used to assess the similarity of perceptions among three radiologists using the Many-Factor Rasch Measurement Version Facets v3.86.0.^6^ Data analysis was performed using STATA v15 Special Edition (Stata Corp, College Station, TX, USA).

## RESULTS

### Demographic characteristics

Descriptive statistics and differences between adolescents with or without pulmonary sequelae after TB demographic characteristics are described in Table 1. There was a difference in the proportion of moderate and severe nutritional status based on the pulmonary sequelae group and without pulmonary sequelae, with 23.5% moderate malnutrition in the non-sequelae group and 25.7% severe malnutrition in the sequelae group. There was a difference in the proportion of comorbidity, which was higher in the autoimmune group by 11.4% in the sequelae group compared to the non-sequelae group.

**Table 1.** Demographic characteristics of adolescent PTB survivors in Hasan Sadikin General Hospital, Bandung, Indonesia, January 2019–December 2022.

Parameters	Pulmonary sequelae (*n* = 35) *n* (%)	Without pulmonary sequelae (*n* = 17) *n* (%)	*P* value (χ^2^)
Sex			
Male	7 (20.0)	7 (41.18)	0.031
Female	28 (80.0)	10 (58.8)	
Age, years			
10–14	11 (31.4)	8 (47.1)	0.306
≥15	24 (68.6)	9 (52.9)	
Nutritional status			
Normal	24 (68.6)	13 (76.5)	
Moderate malnutrition	2 (5.7)	4 (23.5)	<0.0001
Severe malnutrition	9 (25.7)	0 (0.0)	
Monthly family income			
< Minimum wage	31 (88.7)	12 (70.6)	0.036
≥ Minimum wage	4 (11.4)	5 (29.4)	
BCG immunisation			
Yes	33 (94.1)	16 (94.1)	0.154
No	2 (5.7)	1 (5.9)	
Smoking habits per day			
No smoking	33 (94.3)	17 (100)	0.387
Mild (1–10 cigarettes)	1 (2.8)	0 (0)	
Moderate (11–20 cigarettes)	1 (2.8)	0 (0)	
Comorbidity			
None	29 (82.9)	16 (94.1)	
HIV	2 (5.7)	1 (5.9)	<0.013
Autoimmune	4 (11.4)	0 (0)	
TB contact			
Identified	6 (17.1)	5 (29.4)	0.078
Not identified	29 (82.9)	12 (70.6)	
Episode of TB treatment			
None	30 (85.7)	15 (88.2)	
1–2 times	2 (5.7)	1 (5.9)	0.034
>2 times	3 (8.6)	1 (5.9)	
Bacteriological confirmation			
Yes	23 (65.7)	13 (76.5)	0.430
No	12 (32.3)	4 (23.5)	
AFB smear			
Positive	12 (34.3)	2 (11.8)	0.064
Negative	23 (65.7)	15 (88.2)	
Xpert MTB/Rif			
Positive	23 (65.7)	13 (76.5)	0.015
Negative	12 (34.3)	4 (23.5)	
*M. tuberculosis* culture			
Positive	16 (45.7)	5 (29.4)	0.000
Negative	19 (54.3)	12 (70.6)	
Duration from onset to initial therapy, months			
<1	6 (17.1)	4 (23.5)	
1–2	15 (42.9)	4 (23.5)	0.043
>2	14 (40.0)	9 (52.9)	
Duration from completed treatment to evaluation, months, median [IQR]	6 [2–12]	6 [3–11]	0.883
Cut-off duration from completed treatment to evaluation, months, median			
≥6	20 (57.1)	10 (58.8)	0.908
<6	15 (42.9)	7 (41.2)	

PTB = pulmonary TB; BCG = bacille Calmette-Guérin; AFB = acid-fast bacilli; IQR = range interquartile.

There were 283 adolescents who completed treatment or were cured of PTB during the 4-year study period. A total of 52 out of 283 (18.4%) adolescents met the inclusion during the study period. The study included a total of 52 survivors who provided data for analysis. The reasons for exclusion from the study are further underlined in the Figure.

**Figure.**
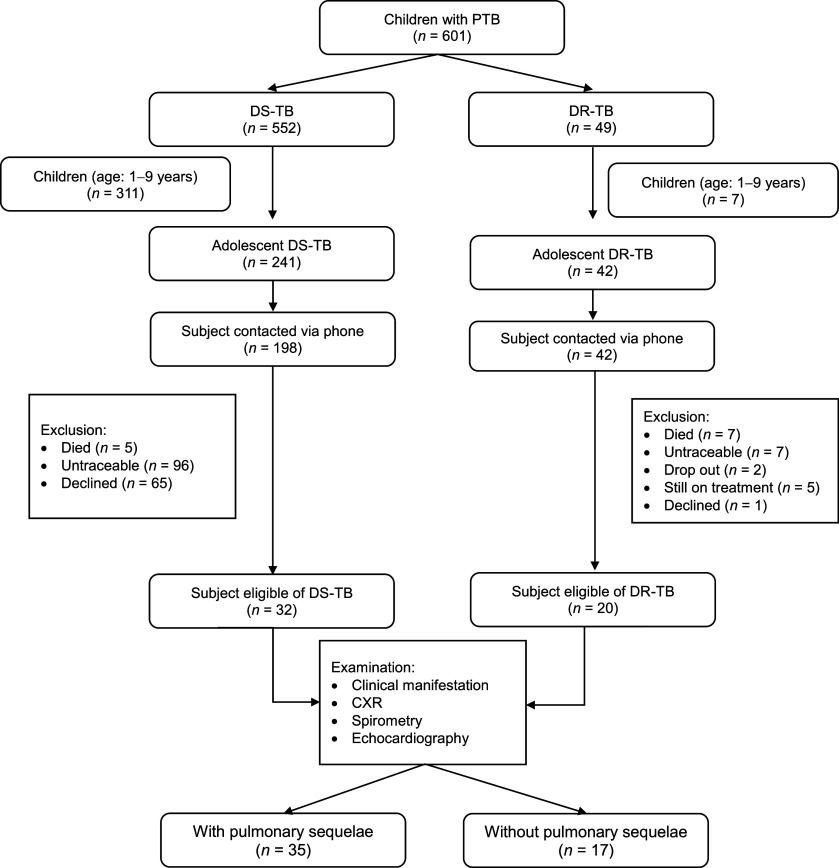
Flow chart for participant recruitment from January 2019 to December 2022. PTB = pulmonary TB; DS-TB = drug-susceptible TB; DR-TB = drug-resistant TB; CXR = chest X-ray.

### Risk factors for pulmonary sequelae after TB in adolescent PTB survivors

Risk factors for pulmonary sequelae after TB are described in Table 2. None of the variables was significant (*P* < 0.05) as risk factors for pulmonary sequelae after TB. However, variables that had *P* < 0.250 were subjected to multivariate logistic regression, and it was found that having a comorbidity had a 1.5 times risk of experiencing pulmonary sequelae compared to those without.

**Table 2.** Risk factors of pulmonary impairment after TB in adolescent PTB survivors.*

Parameters	Pulmonary sequelae (*n* = 35) *n* (%)	Without pulmonary sequelae (*n* = 17) *n* (%)	OR (95% CI)	*P* value <0.05	*P* value <0.250
Age (early adolescent)	11 (57.9)	8 (42.1)	0.796 (0.514–1.231)	0.306	
Female sex	28 (73.7)	10 (26.3)	1.473 (0.844–1.572)	0.173	0.125
DR-TB	16 (80.0)	4 (20.0)	1.347 (0.939–1.932)	0.105	0.148
Family income below minimum regional wages	31 (72.1)	12 (27.9)	1.622 (0.763–3.446)	0.208	0.259
Duration from symptoms to receiving adequate TB treatment >1 month	29 (69.1)	13 (30.9)	1.150 (0.667–1.984)	0.614	
Bacteriologically confirmed PTB	23 (63.9)	13 (36.1)	0.851 (0.585–1.238)	0.402	
History of smoking	2 (100.0)	0 (0.0)	–	–	
Repeated PTB regimen	5 (71.4)	2 (28.6)	1.071 (0.642–1.787)	0.792	
No BCG-vaccinated	2 (66.7)	1 (33.3)	0.989 (0.434–2.255)	0.981	
Malnutrition after TB regimen	11 (73.3)	4 (26.7)	1.130 (0.768–1.663)	0.534	
Had TB contact	6 (54.5)	5 (45.5)	0.771 (0.434–1.369)	0.375	
Had comorbidity	6 (85.7)	1 (14.3)	1.330 (0.916–1.929)	0.133	0.010
Duration from completed treatment to evaluation cut-off	20 (66.7)	10 (33.3)	0.977 (0.667–1.431)	0.908	

* Logistic analysis regression.

PTB = pulmonary TB; OR = odds ratio; CI = confidence interval; DR-TB = drug-resistant TB; BCG = bacille Calmette-Guérin; AFB = acid-fast bacilli.

### Comparison between adolescent DR-TB and DS-TB survivors with pulmonary sequelae after TB

Comparison of clinical manifestations between participants with previous DR-TB and DS-TB were described in Table 3. Of 52 adolescent PTB survivors, 35 (61.5%) adolescents fulfilled the criteria for pulmonary sequelae after TB, with respectively 16 and 19 adolescents with previous DR-TB and DS-TB. Persistent clinical symptoms were reported in 26 of 35 participants (74.3%). All 35 (100%) participants had abnormal CXR results. Of 35 participants, 34 (97.1%) presented with spirometry abnormalities. Using echocardiography TRV assessment, 2 out of 35 (5.7%) participants presented with low risk of PH based on echocardiography results.

**Table 3.** Comparison between adolescent DR-TB and DS-TB survivors with pulmonary impairment after TB.

Parameters	History of DR-TB (*n* = 16) *n* (%)	History of DS-TB (*n* = 19) *n* (%)	*P*-value*
Persistent clinical manifestations (26/35, 74.3%)			
Cough			0.023
Yes	3 (18.8)	8 (42.1)	
No	13 (81.2)	24 (57.9)	
Haemoptysis			0.067
Yes	0 (0)	1 (5.3)	
No	16 (100.0)	18 (94.7)	
Dyspnoea on effort			0.059
Yes	1 (6.3)	2 (10.6)	
No	15 (93.7)	17 (89.4)	
Fatigue			0.016
Yes	8 (50.0)	15 (79.0)	
No	8 (50.0)	4 (21.0)	
Daily activity limitations			0.035
Yes	0 (0)	5 (26.3)	
No	16 (100.0)	14 (73.7)	
Chest X-ray abnormality (35/35, 100%)			
Infiltrates			0.045
Yes	9 (56.3)	9 (47.4)	
No	7 (43.7)	10 (52.6)	
Consolidation			0.073
Yes	2 (12.5)	2 (10.5)	
No	14 (87.5)	17 (89.5)	
Cavity			0.084
Yes	3 (18.7)	3 (15.8)	
No	13 (81.3)	16 (84.2)	
Bronchiectasis			0.056
Yes	0 (0)	2 (10.5)	
No	16 (100.0)	17 (89.5)	
Fibrosis			0.135
Yes	16 (100.0)	14 (73.7)	
No	0 (0)	5 (26.3)	
Pleural thickening			0.257
Yes	10 (62.5)	6 (31.6)	
No	6 (37.6)	13 (68.4)	
Spirometry (34/35, 97.1%)			0.038
Normal	1 (6.3)	0 (0)	
Mild restriction	4 (25.0)	10 (52.6)	
Moderate restriction	6 (37.4)	3 (15.8)	
Severe restriction	4 (25.0)	6 (31.6)	
Moderate–severe obstruction	1 (6.3)	0 (0)	
Echocardiography (2/35, 5.7%)			1
Normal	15 (93.7)	18 (94.7)	
Low risk of PH	1 (6.2)	1 (5.3)	

* Statistically significant <0.05.

DR-TB = drug-resistant TB; DS-TB = drug-susceptible TB; PH = pulmonary hypertension.

A significantly higher prevalence of persistent clinical symptoms was found in the DS-TB group compared to the DR-TB group (*P* < 0.05). Only infiltrates in CXR were found in significantly higher rates in the DS-TB group compared to the DR-TB group (56.3% vs. 47.4%; *P* < 0.05). A higher, non-significant (*P* > 0.05) prevalence of other abnormalities detected by CXR was reported in the DR-TB group. The exact multi-rater agreement of the radiologists was 86.2% (0.862), which is almost perfect. Moderate airway restriction was significantly more prevalent in pulmonary sequelae after TB adolescents with a previous history of DR-TB (*P* = 0.038). Echocardiography assessment revealed that one participant from each of the DR-TB and DS-TB groups showed signs indicative of a low probability of PH.

## DISCUSSION

A higher prevalence of female TB survivors compared to males was found in this study, similar to previous studies.^13–15^ Humayun et al. reported the opposite findings in our study; the aforementioned study had suggested a 53% higher risk of males contracting TB than females.^16^ Conversely, Thakur et al. reported a higher ratio of female adolescents (<19 years) compared to male adolescents for the prevalence of both intra- and extra-PTB.^17^ However, a lower male/female ratio in our study compared to the aforementioned studies may affect the interpretation of PTB prevalence.

Almost a third of adolescent survivors with pulmonary sequelae after TB showed malnutrition after completion of TB treatment (*P* < 0.0001). Malnutrition is a risk factor for TB and vice versa.^18^ Nutritional deficiency may lead to immune dysfunction, increasing susceptibility to TB; simultaneously, PTB infection may cause cachexia, as noted by lower serum albumin concentration compared to healthy controls, due to increased metabolic rate from chronically active immune system against PTB.^19^

Gandhi et al. reported that smoking, education, body mass index (BMI), delayed TB diagnosis, and previous TB treatments were significant risk factors for pulmonary impairment after TB.^20^ In comparison, our study showed that only comorbidity was a significant risk factor for pulmonary sequelae in adolescent PTB survivors. A portion of participants experienced an illness duration exceeding 2 months before initiating adequate therapy, similar to a study by Maior et al. involving Brazilian adolescents with a previous history of PTB.^21^ Conversely, Vecino et al. reported that pulmonary function impairment was not significantly associated with the delay in treatment seeking.^22^

In this study, persistent clinical symptoms in adolescents with pulmonary sequelae after TB were more prevalent in DS-TB cases compared to DR-TB (*P* < 0.05). However, potential social stigma and earlier detection tied with stricter monitoring regimens in Indonesian DR-TB sufferers may potentially cause lower prevalence or reports of clinical symptoms in DR-TB patients.

Studies in adults reported a higher prevalence of radiological abnormalities in DR-TB than in DS-TB.^23–27^ Similar findings using CXR, with infiltrates, were significantly more prevalent in the DR-TB group. In our study, bronchiectasis was found in 10.5% of DS-TB survivors. The DR-TB group had more cavities on CXR compared to the DS-TB; cavities were associated with worse outcomes in TB survivors.^21,27^ Currently, no guidelines have suggested an optimal timing for radiological evaluation of PTB survivors,^8,25,26^ possibly due to variations in the onset of radiological abnormalities in PTB survivors.^26^

In spirometry, adolescents with DR-TB present with a higher prevalence of moderate restrictive patterns compared to mild restriction patterns in the DS-TB group. While the prevalence of pulmonary impairment in adolescents reported in this study is similar to that in adult survivors,^4^ obstructive patterns were less prevalent in this study compared to adults;^28–30^ Restrictive spirometry patterns may be consistent with a higher prevalence of parenchymal damage preceding airway damage;^20,31,32^ although our current study had yet to demonstrate the association between both.

Echocardiographic abnormalities were found in 2 of 52 participants with low risk for PH. Bhattacharyya et al. reported that 15% of adult patients with PH had a history of PTB.^33^ To the best of our knowledge, studies for the correlation of PH in paediatric PTB survivors are currently unavailable. Both cases with low risk for PH had shown extensive and multiple CXR abnormalities. Thus, further studies on the association of PH and CXR abnormalities may be warranted.

This study had several limitations. Of all the determinants, only comorbidities are significant risk factors for pulmonary sequelae, so more subjects are needed. Lack of baseline and serial examination may prevent establishing progression in lung damage and pulmonary impairment after TB. No distinctions were made between pulmonary sequelae after TB among clinically or confirmed DS-TB survivors, despite over one-third of participants (7 of 19, 36.8%) presenting with bacteriologically confirmed DS-TB. The absence of chest high-resolution computed tomography (HRCT) may limit the sensitivity of identifying radiologic abnormalities. However, the radiation risk associated with the examination may outweigh the benefits of added accuracy.^24^ Accuracy in determining TRV might be diminished due to selecting echocardiography instead of cardiac catheterisation^12^ to maintain participants’ compliance. While long-term sequelae of PTB had been recognised in a few guidelines for TB management, such as the WHO End TB Strategy, no diagnostic criteria have been established for complication, causing the lack of concurrency in case definitions between this and prior studies on post-TB pulmonary sequelae.^34^ Despite the limitations, the study results may provide valid and reliable information on pulmonary sequelae after TB in adolescents with DR-TB and DS-TB. The results may be used as a basis for adolescent TB healthcare policies.

## CONCLUSION

This study has documented pulmonary sequelae after TB among adolescents, indicating the necessity of monitoring after treatment completion for all TB survivors. Adolescents with comorbidities may require more rigorous post-TB treatment monitoring to prevent post-TB sequelae and enable their early detection. We recommend that further research be conducted with a larger sample size, employing a multicentre, prospective design, and incorporating selective chest HRCT for survivors with severe lung damage.
